# Music Therapy and Music-Based Interventions in Pediatric Neurorehabilitation

**DOI:** 10.3390/children12060773

**Published:** 2025-06-14

**Authors:** Elisa Milcent Fernandez, Christopher J. Newman

**Affiliations:** 1 Medical School, Lausanne University, 1005 Lausanne, Switzerland; elisa.milcent-fernandez@unil.ch; 2 Pediatric Neurology and Neurorehabilitation Unit, Lausanne University Hospital, University of Lausanne, 1011 Lausanne, Switzerland

**Keywords:** music therapy, pediatric, neurorehabilitation

## Abstract

Background: Music therapy and music-based interventions are increasingly recognized as valuable adjuncts in pediatric neurorehabilitation, leveraging rhythm, singing, instrument playing, and improvisation to support children with neurological disabilities. Objective/Method: This narrative review synthesizes evidence from studies published between 2000 and 2025, focusing on children aged 3 to 18 years receiving neurorehabilitation. Results: The literature demonstrates that music therapy and music-based interventions can improve motor function—particularly gait and upper limb coordination—as well as speech production, while also reducing anxiety and enhancing participation. Techniques such as rhythmic auditory stimulation and melodic intonation therapy have shown promise in targeting movement and communication deficits. Music therapy is further associated with positive effects on vital signs and emotional well-being, supporting its role in holistic care. Neurobiological findings suggest that music-based interventions may promote neuroplasticity and strengthen brain connectivity, though high-quality mechanistic studies remain limited. Conclusions: Despite methodological heterogeneity and small sample sizes in the current literature, the overall evidence supports music therapy and music-based interventions as accessible, cost-effective, and child-centered complements to standard neurorehabilitation. Future research should prioritize rigorous clinical trials and neurobiological investigations to clarify mechanisms and optimize therapeutic protocols.

## 1. Introduction

Music therapy and music-based interventions are established clinical tools that use structured musical interventions to achieve therapeutic goals for a wide range of patient populations [1,2]. Their application in neurorehabilitation supports improvements in communication, mobility, cognition, and emotional well-being for individuals with neurological conditions. Music therapy, with origins stretching back to ancient civilizations, has evolved into a recognized healthcare profession [2,3,4]. The use of music for healing is well documented in the traditions of ancient India and Greece, where it was believed to restore balance and promote well-being. During the Renaissance, figures such as Marsilio Ficino advocated for music listening as a remedy for melancholy. However, it was not until the 20th century—particularly after World War II—that music therapy emerged as a distinct field. Music interventions are termed as music therapy when they are implemented with the aim of deriving therapeutic effects for patients and are delivered by qualified and certified music therapists within a therapeutic relationship. Today, music therapy is practiced globally and is recognized as a complementary therapy within multidisciplinary healthcare settings. According to a 2021 bibliometric analysis, the overall publishing trend in music therapy was positive over the previous two decades [1], showing growing interest in this approach.

Music therapy and music-based interventions incorporate various musical elements, such as rhythm, singing, playing various instruments, and improvisation, to support children during medical treatment. Evidence suggests that music therapy can make hospital experiences less traumatic for children [5,6], boost their motivation, and improve adherence to treatment [7,8,9]. It is used across a wide range of conditions, including psychiatric disorders (e.g., depression, attention disorders), rehabilitation, relaxation, and memory loss.

Neurorehabilitation addresses the needs of children with neurological disabilities—primarily those involving motor impairments—which may be either congenital or acquired. Common conditions include cerebral palsy, neuromuscular disorders (such as myopathies), genetic syndromes (e.g., Rett, Angelman, Down syndrome), spinal cord malformations, or traumatic brain injuries [10]. These neurological conditions can significantly affect a child’s development and autonomy. Among them, cerebral palsy is the most prevalent affecting 1 in 500 neonates [11]. Because these disabilities can have a profound impact on daily functioning and long-term outcomes, comprehensive and timely neurorehabilitation is crucial to support optimal development and independence.

Standard pediatric neurorehabilitation typically includes physiotherapy, occupational therapy, and speech language therapy, coordinated with other pediatric specialties such as orthopedic surgery. The multidisciplinary approach aims to ensure consistent follow-up and care, involving families and community services [12,13]. A key advantage of music therapy is its adaptability to various settings: hospital, outpatient, or home environments. The foundation of this approach lies in neuroplasticity: music is purported to stimulate the development of brain connections and the secretion of hormones and neurotransmitters, leading to improved motor programming and smoother movements [14,15].

Despite growing clinical interest and promising preliminary findings, the evidence base for music therapy and music-based interventions in pediatric neurorehabilitation remains heterogeneous, with considerable variation in study designs, patient populations, intervention protocols, and outcome measures. This narrative review aims to synthesize and critically appraise the current literature on the effectiveness and neurobiological underpinnings of music therapy for children aged 3 to 18 years with neurological disabilities. By consolidating findings across diverse clinical contexts, this review seeks to clarify the scope of music therapy’s benefits, identify gaps in the literature, and inform future research and clinical practice.

## 2. Materials and Method

This narrative review was conducted in accordance with existing recommendations for narrative synthesis in the health sciences literature [16,17]. A systematic research strategy was developed to identify relevant publications on the use of music therapy and music-based interventions in pediatric neurorehabilitation. The following electronic databases were searched: PubMed, CINAHL, and Google Scholar. The research covered literature published from January 2000 through April 2025. The primary search terms included combinations of “music therapy,” “music,” “pediatric,” “children,” “neurology,” “rehabilitation,” and “neurorehabilitation.” Boolean operators were used to optimize search sensitivity and specificity, with the principal search string being: (music therapy OR music) AND (pediatric OR children) AND (neurorehabilitation OR neurology OR rehabilitation). An additional reference published in 1984 was included due to its historical significance and relevance, though it was treated as a secondary source [18].

**Figure 1:**
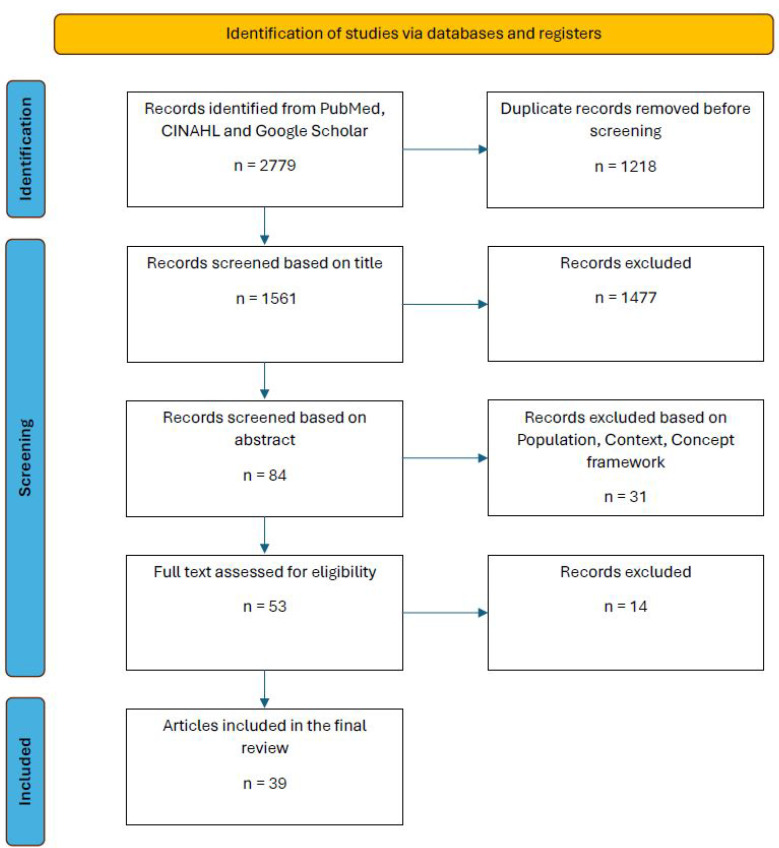
PRISMA Chart.

Study selection was guided by the PCC (Population, Context, Concept) framework. Eligible studies involved children aged 3 to 18 years with neurological disabilities (Population), within the context of pediatric neurorehabilitation settings (Context), and focused on the use of music therapy or music-based interventions as a complementary—not primary—therapeutic modality (Concept). Identified records were managed using Zotero^®^ (Digital Scholar, Vienna, VA, USA). Titles and abstracts were screened for relevance, and full texts of eligible studies were reviewed for inclusion. Reference lists of included articles were examined to identify additional relevant literature. For data extraction and synthesis, a standardized data chart was used to document key characteristics of each included study, such as study design, sample size, intervention type, and outcomes measured. Thematic analysis was employed to identify recurring patterns, principal findings, and knowledge gaps across the selected literature. Cross-referencing enabled comparison of results and facilitated the identification of consistent trends as well as areas requiring further investigation. The process of study selection and inclusion is summarized in Figure 1.

## 3. Results

The effects of music therapy and music-based interventions in pediatric neurorehabilitation were analyzed across several domains, including general benefits, motor function (lower and upper limbs), and speech. The following section synthesizes the main findings from the literature, highlighting the diversity of study designs, populations, and targeted neurological conditions.

### 3.1. Overview of Included Studies and Populations

A total of 39 sources were included in this review, encompassing a wide range of methodologies. Most studies have been published since 2010, reflecting growing interest in the field. While most clinical trials featured small sample sizes (typically 10–25 participants), reviews often thematically synthesized findings from large numbers of studies, providing a broader perspective on the evidence base.

The populations studied primarily comprised children aged 3 to 18 years with neurological disabilities. While some studies included broader age ranges or mixed pediatric and adult populations, their focus remained on pediatric neurorehabilitation. The most frequently addressed conditions were acquired brain injury (including traumatic brain injury) [5,14,19,20,21,22,23,24,25,26,27,28,29], movement disorders [9,19,20,25,27,28,29,30,31,32,33], cerebral palsy [8,19,27,29,34,35,36,37,38,39,40,41] and, to a lesser extent, aphasia [19,20,31,42,43] and rare genetic syndromes such as ceroid lipofuscinosis [29,44]. Notably, several studies also explored the application of music therapy in children with autism [6,9] or epilepsy [9], although these were not the primary focus of most articles. Studies reported a large variety of types of music and of modes of use (Table A1).

**Figure 2:**
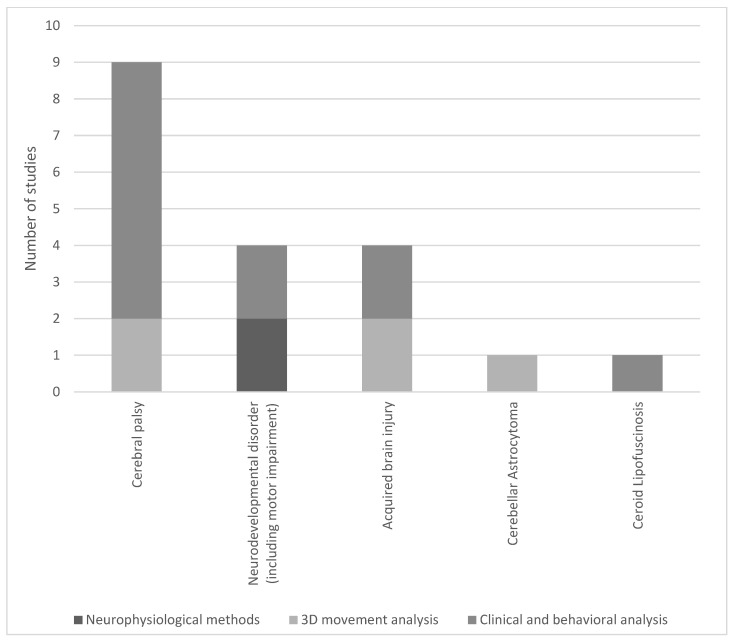
Distribution of conditions and methodological approaches in clinical trials of music therapy.

Twenty clinical trials were identified (Table A2) [5,8,18,20,22,24,25,32,33,34,35,38,39,40,42,44,45,46], with a median sample size of 18 participants (ranging from 1 to 36). They primarily involved children aged from five weeks to 20 years, though one study also included adults [46]. Among them, a majority were quasi-experimental single group trials or single case studies, four were randomized controlled trials, of which two were assessor-blinded, and four were controlled trials without randomization or blinding. For two sources, the control groups were typically developing children [20,35]. The trials employed a variety of assessment tools, such as video analysis, semi-structured interviews, and standardized clinical scores. Five used 3D movement analysis [24,25,36,42,46]. Notably, only a minority incorporated neurobiological methodologies, with two studies utilizing neuroimaging [20,34] and one employing EEG [37], as shown in Figure 2. Intervention was provided by music therapists for 15 of the trials [5,8,18,22,24,25,33,34,35,39,42,44,45,46]. The median duration of the intervention was 8 weeks (4 to 18 weeks). Only seven clinical trials presented longer term results with follow-up after the intervention [8,34,35,38,39,45,47]. Two clinical trials were a one session observation of immediate effects [33,36]. The median follow-up duration was 4 weeks (4 to 16 weeks), with only two above 6 weeks [8,40].

Among the 19 literature reviews [6,9,14,15,19,21,23,26,27,28,29,30,31,37,41,43,48,49,50] a majority were narrative reviews, four were systematic reviews [14,41,47,50], two were scoping reviews [28,29], and one an integrative review [21]. The median number of included references was 33 (ranging from 8 to 69). Reviewed music therapy and music-based interventions varied widely, with the most common techniques being rhythmic auditory stimulation, therapeutic instrumental music performance, and patterned sensory enhancement. Other approaches, such as melodic intonation therapy and singing-based interventions, were primarily reported in the context of speech rehabilitation. The reviewed literature demonstrates both the breadth of music therapy applications in pediatric neurorehabilitation and the heterogeneity of study designs and populations. This diversity highlights the importance of a narrative synthesis to identify consistent trends and knowledge gaps, rather than relying solely on tabular summaries. Subsequent sections will discuss the general and domain-specific effects of music therapy, as well as its neurobiological underpinnings and limitations.

### 3.2. General Effects of Music Therapy and Music-Based Interventions

Eighteen sources described music therapy and music-based interventions as valuable adjuncts to conventional neurorehabilitation for children with neurological disabilities [5,6,7,9,14,15,19,21,22,26,29,30,31,35,47,49]. The structured use of rhythm and melody [14] provided external cues that helped organize therapeutic exercises, reduce distraction [7,40] and foster sustained engagement during sessions. This structured musical environment increased motivation and interest [6,19,22,26,31,35,46,49], which in turn enhanced adherence to therapy and reduced the risk of discontinuation [5,9,21,30,35,46,49].

Beyond motivation and engagement, music therapy and music-based interventions demonstrated a broad range of physiological and psychological benefits for children and adolescents with neurological disabilities [29]. Several studies reported that music therapy and music-based interventions reduced anxiety [5,6,9,15,21,26,31,44], pain, depressive symptoms [6,15,26], and improved overall well-being. These effects are particularly valuable in pediatric populations who often face frequent hospitalizations and intensive therapy regimens, which can be distressing for children and their families [8,9,21,32,34,38,44]. Seven studies, reporting interviews and surveys, highlighted improved interactions not only between the child and their family but also with caregivers and healthcare professionals [6,7,8,9,21,30,38], underscoring music therapy and music-based interventions’ positive impact on the therapeutic environment. However, none of these studies explicitly accounted for children’s experiences, since they mainly relied on proxy reports by caregivers or health professionals. While this could be accounted for by potential difficulties in providing feedback, especially for children with more severe neurological or cognitive impairments, this introduces the risk of observer bias and overall underrepresents children’s subjective experiences.

Importantly, music therapy and music-based interventions had measurable effects on vital signs, reflecting their relaxing and anxiolytic properties. Two studies reported reductions in blood pressure [6,19], five noted decreased heart rate [5,6,8,9,19,21], and five observed lowered respiratory rate [5,6,8,9,19,21]. Three studies also documented increased oxygen saturation during or after music therapy sessions [5,15,21]. Indeed, according to a prospective clinical trial in children with neurodisabilities undergoing rehabilitation [5] heart rate decreased by 8 beats/min, respiratory rate by 0.8 breaths/min and oxygen saturation increased by 0.6% during the use of music therapy. These physiological changes aligned with observable improvements in mental health and comfort, suggesting that music therapy and music-based interventions could support both psychological and somatic aspects of neurorehabilitation.

Music-based interventions are also recognized for their accessibility and cost-effectiveness. Five sources emphasized that they require limited resources and can be implemented in inpatient, outpatient, or home settings, making them a practical addition to standard care [9,15,40,41,48]. Their versatility was further reflected in the diversity of employed techniques. The most common methods in pediatric neurorehabilitation include rhythmic auditory stimulation, therapeutic instrumental music performance, and patterned sensory enhancement. Rhythmic auditory stimulation uses rhythmic cues to facilitate movement execution and control, particularly in gait training [6,14,26,28,30,41]. Therapeutic instrumental music performance involves playing instruments (typically drums or keyboards) to target temporal processing and fine motor skills [8,28,31,32]. Finally, patterned sensory enhancement applies musical elements to guide spatial-temporal and kinematic aspects of daily activities, often used for upper limb rehabilitation [28,33,41].

Finally, the general outcomes of music therapy and music-based interventions encompassed both immediate and long-term effects. One source suggested that music therapy may even influence disease progression in certain chronic conditions [8]. Whether providing acute relief from anxiety or supporting long-term adaptation to chronic illness, music therapy consistently emerged as a flexible, child-centered intervention that enhances the overall effectiveness of neurorehabilitation.

### 3.3. Neurobiological Foundations Supporting the Use and Benefits of Music Therapy and Music-Based Interventions

Twenty-three studies within this review explored neurobiological mechanisms underlying music therapy and music-based interventions’ benefits, including twelve clinical trials and four systematic reviews. While hypotheses about the effects of music on brain function are well-developed, only eight sources provided direct neuroimaging or EEG evidence [14,15,20,27,30,32,34,43,49]. Given this variability in methodological rigor, the level of evidence supporting specific mechanisms is uneven; nonetheless, several convergent themes emerge regarding how music interacts with the developing brain to support neurorehabilitation.

A recurring postulate across fourteen references was that music activates a distributed network of brain regions, including the frontal cortex [15,26,27,32,34,43,49], parietal lobe [15,38], auditory cortex [26,27,30,45], cerebellar networks [22,27,30,35,45], basal ganglia [22], and subcortical structures [14,19,20,32,45]. The involvement of these areas was supported by neuroimaging and electrophysiological studies in a subset of the literature, while other sources extrapolated from behavioral outcomes and known neuroanatomical pathways. For instance, activation of the frontal cortex is associated with motor planning and preparation [15,34], whereas the cerebellum and basal ganglia are implicated in temporal processing and movement coordination [15,19,22]. The arcuate fasciculus, which connects temporal and frontal regions, appears to facilitate the integration of auditory and motor information, a process relevant for both movement and speech functions [19]. Using functional MRI and dynamic causal modeling, Alves-Pinto et al. identified an increase in positive connectivity from the left primary motor cortical area to the right cerebellum in a randomized controlled trial before to after the piano training [20]. Three reviews reported comparisons between musician and non-musician’s brain anatomy, illustrating that music activates brain regions involved in motor planning and execution [19,43,49].

Structural and functional neuroplasticity also featured prominently in the reviewed studies. Evidence from imaging studies in children undergoing music-based interventions, as well as in young musicians, demonstrated increased gray matter density and myelination in motor and auditory regions, alongside enhanced interhemispheric connectivity [19,43]. EEG studies further corroborated these findings, revealing greater coherence in prefrontal and temporoparietal networks following music therapy and music-based interventions [22,30]. Biomarkers such as brain-derived neurotrophic factor (BDNF) [15,30] and mismatch negativity [34] have also been observed to increase in response to musical training, suggesting that music therapy and music-based interventions may drive adaptive synaptic changes, added to the observation of axonal sprouting and enlargement of myelin cells [19,22,43].

Another proposed mechanism was the engagement of the brain’s reward system. While direct pediatric evidence remains limited, some neuroimaging data suggested that music activates regions such as the insula and striatum [19,49], and several studies hypothesized that the release of dopamine and serotonin during music activities enhances motivation, attention, and participation in therapy [6,19,22,26,39].

Finally, ten sources highlighted the principle of rhythmic enhancement, or entrainment [9,15,19,20,22,25,26,27,28,30,32,34,36,39,44,46,47]. Rhythmic external stimulation serves as a temporal and spatial cue to functional movements. These rhythmic cues become anticipatory references on which movement is paced [20,25,28,34,39,46]. Music activates several neuronal networks to induce auditory–motor synchronization from cortical to spinal tracts [30,45]. Therefore, rhythmic auditory stimulation could entrain both cortical and spinal motor circuits, reduce movement variability, and facilitate smoother and more coordinated execution.

In summary, while the strength of evidence varied, the reviewed literature suggested that music therapy and music-based interventions exert their beneficial effects in pediatric neurorehabilitation through multisensory brain activation, promotion of neuroplasticity, engagement of reward pathways, and rhythmic entrainment of motor networks. However, the limited number of studies employing advanced neurobiological methods highlights the need for further research to more precisely delineate these mechanisms in children with a neurodisability.

### 3.4. Applications of Music Therapy and Music-Based Interventions in Pediatric Neurorehabilitation

Music-based interventions have been applied across a spectrum of functional domains in pediatric neurorehabilitation, with the most extensively studied outcomes relating to motor function—particularly gait and lower limb mobility. The literature also addressed upper limb function and speech rehabilitation.

#### 3.4.1. Gait and Lower Body Function

**Figure 3:**
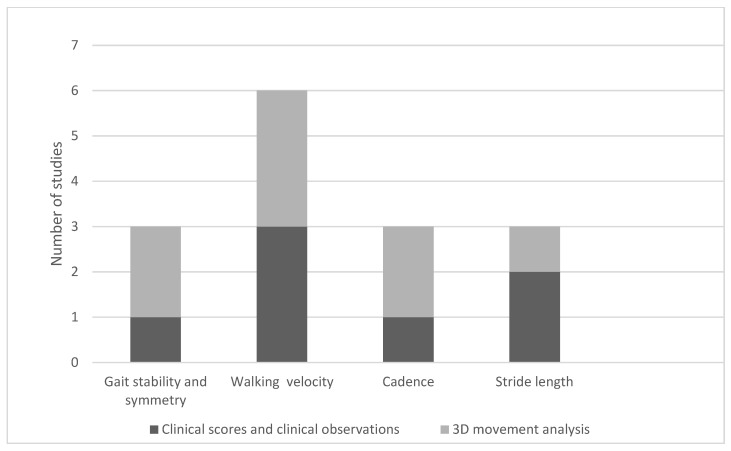
Distribution of improvements in gait parameters and assessment methods in clinical trials.

Eighteen studies in this review evaluated the impact of music therapy and music-based interventions on lower body function, including eight clinical trials [22,24,33,38,42,45,46,47] and ten literature reviews [9,20,21,26,27,30,31,38,45]. The methodologies employed varied, encompassing clinical scoring systems, standardized questionnaires, video analysis [22,24,36,45], and advanced motion capture techniques such as 3D gait analysis [19,25,26,33,45] (Figure 3).

Across these studies, the most widely used music therapy and music-based intervention was rhythmic auditory stimulation [17,21,26,30,42,46], with some studies also employing patterned sensory enhancement [22,33,46] and therapeutic instrumental music performance [22]. The primary gait parameters assessed included walking velocity, cadence, stride length, swing time, and overall stability.

Most studies reported significant improvements in gait function following music therapy and music-based interventions. Sixteen out of eighteen articles concluded that music therapy and music-based interventions, particularly when rhythm-based techniques such as rhythmic auditory stimulation were used, led to measurable gains in walking velocity [19,21,22,23,24,25,26,30,31,33,36,42,45,46], cadence [21,24,25,26,30,42,45,46], and stride length [19,22,26,30,45,46]. Improvements in gait stability [15,24,26,27,29,38,45] were documented in five studies, while enhanced movement regularity and symmetry were also commonly observed [19,26,27,30,36,45,46]. Notably, rhythmic auditory stimulation was associated with immediate and clinically meaningful changes in gait speed and temporal variability, supporting its use as a practical adjunct to conventional rehabilitation [19].

However, the evidence was not uniformly positive. Two studies did not find significant effects of music therapy and music-based interventions on gait parameters [40,41], and the impact on swing time was less consistently reported. Moreover, some studies suggested that the degree of improvement may depend on the specific technique employed, with rhythmic auditory stimulation generally outperforming patterned sensory enhancement in increasing walking velocity.

Beyond spatiotemporal gait parameters, several studies examined joint kinematics and angular displacement at the hip [19,25,38,40], knee [25,33,40,46], and ankle [46]. Music therapy was associated with increased joint range of motion and smoother, more efficient movement patterns. In the randomized controlled trial of Kim et al. [25], the use of rhythmic auditory stimulation improved joint range of motion during gait from 54° to 60° for the hip and from 72° to 78° for knee flexion. These biomechanical improvements were corroborated by further motion analysis data, which showed enhanced spatial and temporal coordination during gait cycles [19,22,25,45].

Importantly, music therapy and music-based interventions’ benefits extended to functional tasks beyond walking. For example, studies utilizing patterned sensory enhancement documented improvements in loaded sit-to-stand exercises, with children demonstrating increased strength and endurance when rhythmic cues were provided [33,40,42]. Patterned sensory enhancement reduced the time required to execute loaded sit-to-stand from 2.33 s to 1.92 s [33]. In this randomized controlled trial, this observation was accompanied by an increase in total knee extension. Furthermore, according to a second study, the intervention group undergoing patterned sensory enhancement over the course of 6 weeks showed a significantly higher increase in load every two weeks compared to control group, respectively, at 6.1 to 7.6 to 8.8 kg versus 5.7 to 6.4 to 7.0 kg [40]. These findings suggest that music therapy and music-based interventions not only optimize gait, but also facilitate broader aspects of lower limb function relevant to daily activities.

In summary, the available evidence supports the integration of music therapy and music-based interventions—especially rhythm-based modalities—into pediatric neurorehabilitation programs targeting lower body function. While the heterogeneity of study designs and outcome measures precludes definitive conclusions, the consistency of positive findings across multiple methodologies underscore the potential of music therapy and music-based interventions as valuable and accessible tools for enhancing motor recovery in children with neurological disabilities.

#### 3.4.2. Upper Limb Function

**Figure 4:**
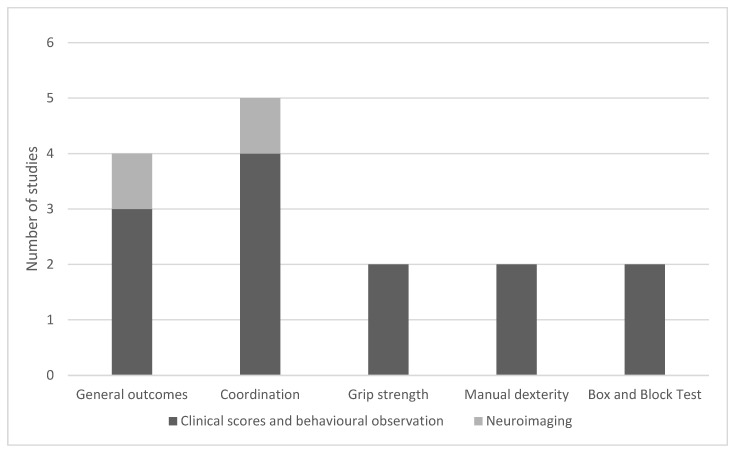
Distribution of improvements in upper extremity function and assessment methods in clinical trials.

Twelve sources in this review examined the effects of music therapy and music-based interventions on upper body function in children undergoing, encompassing eight clinical trials [8,19,20,32,35,38,39,40] and six literature reviews [19,21,26,28,31,41]. The majority of these studies assessed outcomes using standardized clinical scores, such as the box and block test [32,35] for manual dexterity, grip strength measurements, and observational assessments of gross and fine motor skills (Figure 4). Two studies also incorporated neuroimaging (fMRI) to investigate underlying neural mechanisms [20,32].

The employed interventions varied across studies, with rhythmic auditory stimulation [20,21,26,35], therapeutic instrumental music performance [31,32], and patterned sensory enhancement [47] being the most frequently applied techniques. Some studies focused exclusively on a single method, while others compared the effects of multiple music therapy modalities, including singing and melodic intonation therapy.

Overall, eight out of thirteen sources reported general improvements in upper limb function and movement quality following music therapy interventions. These improvements included enhanced manual dexterity [20,21,31,44], increased grip strength [8,14,31,44], and better hand coordination [8,20,31,32,38,40], particularly when instrument-based or rhythm-driven approaches were used. According to a quasi-experimental study, improvement was greater with therapeutic instrumental music performance, hypothetically associated with more intense temporal processing [8]. Piano-based interventions (therapeutic instrumental music performance) were associated with significant gains in finger dexterity and bilateral hand function, as demonstrated by improved performance on the box and block test [35,40] and corroborated by fMRI evidence of increased recruitment of multimodal brain areas involved in motor control [8,32]. While keyboard playing increased fine motor skills and dexterity, playing drums lead to improvements in reach, grasp, coordination, and grip strength [19,21,31].

However, the evidence was not uniformly positive. Five studies did not observe statistically significant improvements in upper limb function compared to standard rehabilitation alone [26,35,38,40,44]. These discrepancies may be attributed to differences in study design, intervention duration, or the severity of participants’ motor impairments. Notably, while gross motor function did not consistently improve across all studies [26,40], reductions in movement time variability and enhanced movement speed were frequently observed, suggesting that music therapy and music-based interventions may optimize the efficiency and timing of upper limb movements even in the absence of large gains in strength or range of motion.

An interesting finding reported by three studies was that the non-dominant hand, or the hand with poorer baseline motor function, tended to benefit most from music therapy and music-based interventions [20,32,35,40]. This was reflected in greater improvements in dexterity and coordination, particularly following keyboard-based exercises [32,35].

In summary, while the current literature suggests that music therapy and music-based interventions can support upper limb rehabilitation in children with neurological disabilities—especially in terms of timing, coordination, and fine motor skills—the magnitude and consistency of these effects remain variable.

#### 3.4.3. Speech

Thirteen sources in this review addressed the application of music therapy and music-based interventions in speech rehabilitation for children with neurological disabilities, including eleven literature reviews [9,14,19,21,26,27,30,31,43,48,49] and two clinical trials [18,44]. The principal techniques examined were melodic intonation therapy and singing-based interventions, both of which leverage the musical and rhythmic aspects of language to facilitate speech production and fluency, particularly in children with aphasia or motor speech disorders.

Melodic intonation therapy, which utilizes melodic intonation and rhythmic tapping to support the articulation of words and phrases, was consistently reported as beneficial for children with non-fluent aphasia [14,19,26,43,48]. Five articles specifically highlighted its effectiveness, with one reporting an approximate 20% improvement in spontaneous speech production following intervention [26]. The underlying rationale is that singing and rhythmic intonation engage preserved neural pathways in the right hemisphere, which can compensate for damaged language areas in the left hemisphere [19]. Neuroimaging studies included in the review corroborated this mechanism, demonstrating that music-based interventions activate brain regions associated with both language and motor planning [14,22,43], and may promote the development of alternative neural networks for speech. Indeed, based on previous studies of musicians’ brains, one review highlighted that music remodeled the arcuate fasciculus through changes in myelination, axon diameter, and axonal sprouting [43]. This structure is essential for auditory-motor mapping and therefore language development.

Beyond melodic intonation therapy, singing exercises—both structured and improvisational—were found to enhance speech fluency by supporting syllable lengthening, reducing speech rate [30,31,43,49], and improving motor mapping and feedback control. These interventions also fostered greater engagement and enjoyment during therapy, which are important for sustained participation and progress. Some studies noted that techniques such as left-hand tapping [19,20,43], synchronized with verbal output, further facilitated the coupling between motor and speech functions, aiding in the rehabilitation process.

In addition to improvements in expressive language, music therapy was associated with enhanced respiratory and phonatory control. Singing, particularly a cappella, was shown to increase inspiratory and expiratory pressures, extend phonation time, and improve conversational voice volume [8,14,30,48]. These physiological benefits are particularly relevant for children with neuromotor speech impairments, as they support the foundational skills required for effective verbal communication.

Overall, the literature supported the use of music therapy, and especially melodic intonation therapy and singing-based approaches, as valuable adjuncts in the speech rehabilitation of children with aphasia and related disorders. The convergence of clinical, neurophysiological, and patient-reported outcomes underscores the promise of music-based interventions in this domain.

## 4. Discussion

This narrative review synthesizes current evidence supporting music therapy and music-based interventions as complementary interventions in pediatric neurorehabilitation, highlighting its physiological, psychological, and neurobiological effects. While the findings are promising, several limitations and priorities for future research must be acknowledged.

Music therapy and music-based interventions demonstrate measurable benefits in three principal domains: gait rehabilitation, upper limb function, and speech recovery. In motor rehabilitation, techniques such as rhythmic auditory stimulation and related approaches have been shown to improve gait parameters, including velocity, cadence, and stride length, by leveraging the brain’s neuroplastic potential [19,22,25,26,28,30,33,36,37,38,42,45,46]. Neuroimaging studies have corroborated that rhythmic cues enhance connectivity between sensory and motor regions, resulting in more efficient movement planning and execution. In upper limb rehabilitation, instrument-based interventions have been linked to improved manual dexterity and bilateral coordination, with evidence of strengthened neural pathways. For speech rehabilitation, melodic intonation therapy and singing-based interventions engage alternative neural networks, compensating for damaged language areas in children with aphasia.

A key mechanism underlying these effects is the enhancement principle [15,19,22,25,27,28,30,38,46,47], in which rhythmic entrainment synchronizes neural oscillations with external auditory cues, reducing movement variability and optimizing motor output. Electrophysiological evidence [22,30] links this process to the activation of central pattern generators in the spinal cord, enabling smoother and more coordinated movements.

Beyond motor and speech outcomes, music therapy and music-based interventions address important psychological and systemic aspects of pediatric neurorehabilitation. By stimulating dopamine and serotonin release [6,19], music enhances motivation, reduces anxiety [5,9,31,38,44,50], and improves adherence to therapy, all of which are crucial for long-term recovery. Physiological markers such as a reduced heart rate [5,6,9,19,32,44] respiratory rate [6,9,19,21,44], and blood pressure [6,19] during music-based interventions further support its anxiolytic effects. Additionally, music therapy and music-based interventions foster positive interactions between children, caregivers, and families [5,7,8,21,26,30,32,34,38,44], helping to mitigate the psychological burden of chronic disability and frequent hospitalizations.

Despite these promising results, the evidence base is limited by several methodological challenges. Many studies suffer from small sample sizes and heterogeneous populations [19,21,24,26,29,31,34,41,42,43,44], sometimes including both pediatric and adult participants [31,44], which restricts the generalizability of findings. Only a minority of the trials were randomized, and of these, just two were assessor-blinded, underscoring significant opportunities for methodological improvement in future studies. The neurobiological mechanisms, while theoretically compelling, are insufficiently explored: only a minority of studies utilized neuroimaging or EEG, and none directly measured neurotransmitter changes in pediatric populations. Furthermore, outcomes vary due to differences in intervention protocols, patient characteristics, and clinical settings [8,31,36,43]. The lack of long-term follow up was common to all included clinical trials. Indeed, only eight trials included observation after the duration of the intervention [8,32,34,35,38,39,40,45], and none above six weeks. Therefore, no conclusions can be drawn on the longer-term effects of music-based interventions. Not every study used music therapy with a certified therapist. Although 29 sources specifically reported the involvement of a music therapist, several reviews did not clearly distinguish between outcomes from music-based interventions and those from music therapy in their descriptions. Notably, one source emphasized that having a credentialed music therapist deliver the intervention is crucial for maximizing treatment outcomes [36]. Further research is warranted to directly compare the effectiveness of music-based interventions conducted with and without the presence of a certified therapist. While the studies included according to the selection criteria primarily focus on neurologic music therapy, it is important to recognize that other forms of music therapy exist and merit further exploration.

Future research should address these limitations by conducting rigorous neurobiological studies using advanced imaging and biomarker analysis, standardizing intervention protocols to facilitate cross-study comparisons, and implementing large-scale randomized controlled trials with well-defined pediatric cohorts and outcome measures. Longitudinal studies are also needed to assess the durability of benefits and the potential for disease modification in progressive neurological conditions.

## 5. Conclusions

This review highlights music therapy and music-based interventions’ multifaceted role in pediatric neurorehabilitation, bridging motor, cognitive, and emotional domains through mechanisms rooted in neuroplasticity and rhythmic entrainment. While current evidence supports its integration into multidisciplinary care, the field is constrained by a lack of high-quality neurobiological research and standardized methodologies. Future studies must prioritize mechanistic investigations and robust clinical trials to validate music therapy’s therapeutic potential, optimize its application, and secure its position as an evidence-based intervention in pediatric neurorehabilitation.

## Data Availability

No new data were created during this study.

## Appendix A

**Table A1:** Types of music and modes of use in included references (PSE: patterned sensory enhancement; RAS: rhythmic auditory stimulation; TIMP: therapeutic instrumental music performance).

Source	Type of Music Used	Active or Passive Music Intervention	Improvisation or Registered Music	Individual or Group Intervention	Use of Music Therapist/Musician/Educator
Altenmüller et al. (2013) [19]	Instrumental and rhythmic	Active	Registered	Both	Music Therapist
Alves-Pinto et al. (2015) [20]	Piano Training—TIMP	Active	Registered	Individual	Educator
Atkinson (2022) [44]	Not specified	Active	Improvisation	Individual	Music Therapist
Baldisseri et al. (2022) [48]	AI-integrated music	Active	Registered	Individual	Not specified
Bower et al. (2021) [14]	Various (review)	Both	Both	Both	Music Therapist
Bringas et al. (2015) [34]	Music Therapy	Active	Registered	Group	Music Therapist
Burns et al. (2025) [21]	Music and Music Therapy	Both	Both	Both	Music Therapist
Cassidy (2020) [22]	Neurologic Music Therapy	Active	Registered	Individual	Music Therapist
Devlin et al. (2019) [30]	Music Therapy, Music-Based	Both	Both	Both	Music Therapist
Dogruoz Karatekin et al. (2021) [35]	TIMP	Active	Registered	Individual	Not specified
Gilbertson (2009) [23]	Music Therapy	Both	Both	Both	Music Therapist
Jensen (2009) [36]	RAS	Active	Registered	Individual	Music Therapist
Kelly et al. (2020) [24]	RAS	Active	Registered	Individual	Music Therapist
Kennelly et al. (2001) [31]	Varied, therapeutic music	Both	Both	Both	Music Therapist
Kim et al. (2010) [42]	RAS	Active	Registered	Individual	Music Therapist
Kim et al. (2016) [25]	RAS	Active	Registered	Group	Music Therapist
Kobus et al. (2022) [5]	Music Therapy	Active	Registered	Both	Music Therapist
Koshimori et al. (2019) [49]	Music based interventions varied	Both	Both	Not specified	Not specified
Kwak (2007) [45]	RAS	Active	Registered	Individual	Music Therapist
Kwak et al. (2013) [37]	RAS	Active	Registered	Group	Music Therapist
Lampe et al. (2015) [32]	Piano Training—TIMP	Active	Registered	Individual	Educator
Liuzzi et al. (2024) [38]	Euterpe Method (custom musical cues)	Active	Registered	Group	Music Therapist
Magee et al. (2017) [26]	Music Interventions	Both	Both	Both	Music Therapist
Marley (1984) [18]	Music	Passive	Registered	Individual	Music Therapist
Marrades-Caballero et al. (2018) [39]	Neurologic Music Therapy	Active	Registered	Group	Music Therapist
Mrázová et al. (2010) [50]	Music Therapy	Both	Both	Both	Music Therapist
Peng et al. (2011) [33]	Therapeutic Music	Active	Registered	Individual	Not specified
Preti et al. (2011) [7]	Live Music Program	Passive	Registered	Group	Musician
Sampaio (2023) [6]	Various (hospital program)	Both	Both	Both	Musician/Educator
Santonja-Medina et al. (2022) [8]	Neurologic Music Therapy	Active	Registered	Group	Music Therapist
Schlaug et al. (2010) [43]	Singing	Active	Improvisation	Individual	Music Therapist
Shin et al. (2015) [46]	RAS	Active	Registered	Individual	Music Therapist
Stegemann et al. (2019) [9]	Various (overview)	Both	Both	Both	Music Therapist
Thaut (2010) [15]	Neurologic Music Therapy	Both	Both	Both	Music Therapist
Thaut et al. (2014) [27]	Rhythmic Entrainment	Active	Registered	Not specified	Music Therapist
Tramontano et al. (2021) [28]	Various (scoping review)	Both	Both	Both	Both
Twyford et al. (2025) [29]	Music Therapy	Both	Both	Both	Music Therapist
Wang et al. (2013) [40]	PSE	Active	Registered	Home-based/Individual	Not specified
Yanagiwara et al. (2022) [41]	Music Therapy	Both	Both	Both	Music Therapist

**Table A2:** Methods and outcomes of included clinical trials.

Source	Intervention	Design	Duration	Outcome Measures	Main Results
Alves-Pinto et al. (2015) [20]	Individual piano training in children with neurodevelopmental disorders by music teacher	Non-randomized [35] clinical trial; intervention group *n* = 10, control group (no intervention) *n* = 6; age: 8–17 years	10-week intervention; no follow-up	fMRI, dynamic causal modeling	Increase in positive connectivity from left primary motor cortical area to right cerebellum from before to after training
Atkinson (2022) [44]	Individual neurologic music therapy in children with ceroid lipofuscinosis by music therapist	3-phase mixed methods single arm study; *n* = 20; age: 3–17 years	12-week intervention; follow-up not reported	Interviews, behavioral observations, functional scales	Improvements in global function and expressive communication
Bringas et al. (2015) [34]	Auditory Attention plus Communication protocol in groups of 4 children with severe neurological disorders by music therapist	Non-randomized clinical trial; intervention group *n* = 17, control group (standard neurorestoration program) *n* = 17; age: 3–12 years	12-week intervention; 4-week post-treatment follow-up	Neuroimaging, clinical scores, EEG	Improved attention and communication as well as changes in brain plasticity
Cassidy et al. (2020) [22]	Rhythmic auditory stimulation, patterned sensory enhancement and therapeutic instrumental music performance in adults and children with acquired brain injury, individually by music therapist	Multiple-baseline single case design; *n* = 20; age: 6–18 years	6-week intervention; no follow-up	10 min walk tests with wearable inertial sensor and video analysis, mood assessments, participant survey	Increase in gait velocity (+8%) and stride length (+10%), large reduction in anxiety, improved mood, increased motivation and engagement levels
Dogruoz Karatekin et al. (2021) [35]	Therapeutic instrumental music performance (piano) in teenagers with cerebral palsy, individually, unspecified interventionist	Non-randomized clinical trial; intervention group *n* = 9, control group (age-matched participants with normal development, same intervention); age: 11–18 years	8-week intervention; 4-week post-treatment follow-up	Clinical motor scores	Gains in grip strength, strengths of the fingers, gross and fine motor skills
Jensen (2009) [36]	Rhythmic auditory stimulation in children with spastic diplegic cerebral palsy	Single case design; *n* = 5; age: 7–13 years	Immediate entrainment study, single sessions	3D gait analysis and video analysis	Improvement in gait symmetry, movement and timing variability
Kelly et al. (2020) [24]	Baseline A—standard rehabilitation (10–30 min physiotherapy/week), Baseline B—intervention with Rhythmic Auditory Session replacing 2/10 of baseline A in children with acquired brain injury	Multiple baseline single case experimental design; *n* = 4; age: 10–13 years	4-week intervention; no follow-up	3D gait analysis and 10 m walk test	Gain in cadence and step symmetry
Kim et al. (2010) [42]	Nine sessions of rhythmic auditory stimulation, 30 min each in child with cerebellar astrocytomas	Single case study; *n* = 1; age: 12 years	8-week intervention no follow-up	3D gait analysis	Gain in gait coordination
Kim et al. (2016) [25]	Rhythmic auditory stimulation 3 ×/week in adolescents with acquired brain injury	Randomized controlled trial; intervention group *n* = 6, median 13.3 years; control group (standard gait training) median age 14.2 years	6-week intervention; no follow-up	3D gait analysis	Gain in stride length, step time, walking speed Gain in joint range of motion during gait: hip 54° to 60° and knee 72° to 78°
Kobus et al. (2022) [5]	Live music therapy during physical therapy 2 ×/week, and two more physical therapy sessions/week without music therapy in children with neurological diseases	Single case design; *n* = 17; age = 0–18 years, median = 3 years	10-week intervention; follow-up not reported	Therapy reports, behavioral response	Changes in vital parameters: mean heart rate decrease of 8 beats/min, respiratory rate decrease of 0.8 breaths/min, O2 saturation increase of 0.6% Improvement in general physical outcomes, decrease in anxiety
Kwak et al. (2007) [45]	Rhythmic auditory stimulation in walking children with spastic cerebral palsy. A music therapist was present for both intervention groups but just observed for the self-guided training group	Three group clinical trial (randomization not described); therapist-guided training group *n* = 9, self-guided training group *n* = 7, control group *n* = 9 (conventional gait training; age: 6–20 years	4-week intervention; 2-week follow-up	Gait performance metrics by stride analysis with heel switches	Significant gains in stride length (+30%) and gait velocity (+37%) in therapist-guided training group
Lampe et al. (2015) [32]	Piano training 35–40 min 2 ×/week with a professional piano teacher in children with hand motor impairments	Single case design; *n* = 18; age: 6–16 years	18-week intervention; no follow-up	Hand function scores, movement observation	Improvements in fine motor control: Box and Block Test improvement of 5.1 and 3.4 blocks for non-dominant and dominant hands
Liuzzi et al. (2024) [38]	Euterpe method 3 times/week with music therapist in children with cerebral palsy during inpatient rehabilitation program	Randomized control trial; intervention group *n* = 25, control group *n* = 10 (standard inpatient rehabilitation without music therapy); Age = 0–10 years	6-week intervention; 2-week follow-up	Motor assessments, clinical scores and questionnaires, parental report Euterpe method: association between music and sensory stimulation	In intervention group additional gain in gross motor function, gait performance and stability Gain in patient’s sleep and quality of life Gain in social interactions observed by parents
Marley et al. (1984) [18]	Music program by music therapist in hospitalized infants and toddlers: relaxation, didactic games, movement and songs. 15 min to 1 h per session	Single case design; *n* = 27; age: 5 weeks to 36 months	8-week intervention; follow-up not mentioned	Behavioral observations	Decreased anxiety Gain in vocalization and sound initiation
Marrades-Caballero et al. (2018) [39]	Therapeutic instrumental music performance in addition to usual physiotherapy for children with severe cerebral palsy	Randomized control trial, assessor-blinded; intervention group *n* = 18, control group *n* = 9 (usual therapeutic input); age: 4–16 years	16 weeks; 4-week follow-up	Chailey levels of ability	Gains in arm-hand position and activities
Peng et al. (2011) [33]	Patterned sensory enhancement during loaded sit-to-stand individually composed by music therapist in children with spastic diplegic cerebral palsy	Single case design; *n* = 23; age: 5–12 years	1 session (immediate effect)	Sit-to-stand metrics	Gain in sit-to-stand speed (2.33 s in control condition, 1.92 s in intervention condition) Gain in load control with increased total knee extensor power and stability
Preti et al. (2011) [7]	Music program for inpatients in a hospital, including improvisation, vocal skills, group performance	Qualitative study: direct observation children *n* = 162, caregivers *n* = 146; interviews children *n* = 14, caregivers *n* = 22; children’s age: 0–16 years	12-week observation; no follow-up	Thematic analysis of direct observations, videos and interviews	Decreased anxiety and increased emotional responses reported by caregivers
Santonja-Medina et al. (2022) [8]	Therapeutic instrumental music performance in children with severe bilateral palsy (percussion, 13 sessions)	Quasi-experimental single group study; *n* = 17; age = 4–18 years	4-month pre intervention (usual therapies); 4-month intervention; no follow up	PEDI-CAT scores, therapist observational reports	Gains in grasping, reaching and hitting with the hand
Shin et al. (2015) [46]	Rhythmic auditory stimulation with gait training in hemiplegic participants with either stroke or cerebral palsy (30 min per session, 3 ×/week)	Single case design; *n* = 18; no age limitation	4-week intervention; no follow-up	3D gait analysis (stride length, cadence)	Gain in stride length and cadence Gain in gait deviation index from 83.9% to 87.3% after intervention
Wang et al. (2013) [40]	Home-based patterned sensory enhancement during loaded sit-to-stand exercises in children with spastic diplegic cerebral palsy	Randomized control trial, assessor-blinded; intervention group *n* = 18, control group (sit-to-stand training without music) *n* = 18; age: 5–13 years	6-week intervention; 12-week follow-up	Clinical scores, clinical observation	Significantly higher gain in GMFM scores in PSE group Mean load increase was stronger in PSE group: 6.1 to 7.6 to 8.8 kg compared to 5.7 to 6.4 to 7 kg every 2 weeks
